# Ionic Liquids-Based Nanocolloids—A Review of Progress and Prospects in Convective Heat Transfer Applications

**DOI:** 10.3390/nano11041039

**Published:** 2021-04-19

**Authors:** Alina Adriana Minea, S. M. Sohel Murshed

**Affiliations:** 1 Faculty of Materials Science and Engineering, Technical University Gheorghe Asachi from Iasi, Bd. D. Mangeron No. 63, 700050 Iasi, Romania; 2 Centre for Innovation, Technology and Policy Research (IN+), Department of Mechanical Engineering, Instituto Superior Técnico, University of Lisbon, 1049-001 Lisbon, Portugal

**Keywords:** ionic liquid, nanoparticles, convection, heat transfer, experimental correlations, thermophysical properties

## Abstract

Ionic liquids are a new and challenging class of fluids with great and tunable properties, having the capability of an extensive area of real-life applications, from chemistry, biology, medicine to heat transfer. These fluids are often considered as green solvents. Several properties of these fluids can be enhanced by adding nanoparticles following the idea of nanofluids. These ionic liquids-based nanocolloids are also termed in the literature as ionanofluids or nanoparticles-enhanced ionic liquids. This review summarizes the findings in both areas of ionic liquids and ionic liquids nanocolloids (i.e., ionic liquids with nanoparticles in suspension) with direct applicability in convective heat transfer applications. The review presents in a unified manner the progress and prospects of ionic liquids and their nanocolloids from preparation, thermophysical properties and equally experimental and numerical works. As the heat transfer enhancement requires innovative fluids, this new class of ionic liquids-based nanocolloids is certainly a viable option, despite the noticed drawbacks. Nevertheless, experimental studies are very limited, and thus, extensive experiments are needed to elucidate ionic liquids interaction with nanoparticles, as well as their behavior in convective heat transfer.

## 1. Introduction

Ionic liquids (ILs) are considered as a candidate for heat transfer applications particularly when nanoparticles are dispersed into them making a new class of fluids (known as ionanofluids) with improved thermal performance. Thus, it is important to briefly highlight thermophysical properties of ILs such as density, viscosity, thermal conductivity and specific heat and how these properties are influenced by temperature and pressure, which are particularly crucial for convective heat transfer application. Similar to common molecular liquids, density of ILs slightly decreases (mostly linearly) with increasing temperature. For instance, at atmospheric pressure, an increase in temperature from 288 to 363 K decreases the density of [BMIM][NTf_2_] from 1446 to 1375.7 kg/m^3^ (4.86%) [1,2]. Density of ILs also changes with pressure, and it increases with increasing pressure. For example, at 298 K density of [BMIM][NTf_2_] increased from 1436 to 1561.5 kg/m^3^ (8.74%) due to increasing pressure from 0.1 (atmospheric) to 249.6 MPa [1]. Although viscosity of ILs is generally higher than those of common heat transfer fluids such as ethylene glycol similar to any other liquids’ viscosity of ILs also decrease considerably (non-linearly) with increasing temperature (e.g., Ferreira et al. [3]). Such a decrease in viscosity of IL is particularly important for the convection applications at elevated temperature, as it can significantly reduce the pumping power. However, unlike conventional liquids, thermal conductivity of ILs was found to decrease slightly (for some ILs almost independent of temperature) with increasing temperature [3,4]. However, specific heat of ILs shows behavior similar to common viscous fluids such as ethylene glycol, as this property increases mostly linearly with temperature [5], which is good for thermal energy storage.

Apart from above properties and their dependent on temperature and pressure, ILs are thermally stable up to considerably high temperature (e.g., 450 °C). Because of interesting characteristics and properties as well as potential applications some special types of ILs such as imidazolium-based ILs are widely used and studied types. Based on application as for convective heat transfer, the properties and features of ILs play an important role for their own performance as well as their nanofluids (INF).

Adding nanoparticles to ionic liquids came as a logical step to increase their thermal conductivity, which is rather low if compared to several well-known heat transfer fluids. A comprehensive discussion on this topic was attained by Minea [6], where the advantages and disadvantages of using ionic liquids for different applications based on the ionic liquids description at molecular level can clearly be noticed. Additionally, Minea [6] discusses thermophysical properties of ionic liquids in comparison with regular heat transfer fluids, outlining both their benefits and drawbacks, concluding that ionic liquids are superior to basic heat transfer fluids mostly in relation of stability, low vapor pressure and environmental safety. The most important feature of ILs is their easy-to-design properties by merging anions and cations, and the most significant feature that distinguishes ionic liquids among regular commercial heat transfer fluids is the extraordinarily low saturated vapor pressure at high temperature. From the state-of-the-art literature, it is obvious that the thermal conductivity is increasing by adding solid nanoparticles to ionic liquid, and the phenomenon occurring is similar with the one observed for nanofluids. In regard to viscosity, a general conclusion is that the viscosity is increasing by adding nanoparticles and is decreasing at heating. More details about this behavior and the changes in thermophysical properties of a number of ionic liquids studied in the open literature can be found in a previous work published by these authors (Minea and Murshed [7]).

One of the first reviews on ionic liquids-based nanocolloids came from Marsh et al. [8] who presented the net advantages of adding nanoparticles to ionic liquids and also discussed their possible applications.

Many papers discuss heat transfer applications: For example, a study performed by França et al. [9] demonstrated that these new fluids, due to high thermal conductivity and specific heat, are suitable candidates for heat transfer applications in a shell and tube heat exchanger. The same conclusion was also reached by other authors (see, for example, [7,10,11,12,13,14,15]) that performed mainly numerical studies on heat transfer performance. Anyhow, it was noticed from the archived literature that, at least by these authors’ knowledge, the number of experimental studies are scarce.

This review came as a continuation of our work, and it summarized and discussed comparatively recent research performed both in the area of ionic liquids and ionic liquids-based nanocolloids, with emphasis on both of these fluids’ thermophysical properties in relation to their convection heat transfer. Nevertheless, the last parts are dedicated to numerical studies performed until now as well as proposed analytical and numerical correlations on heat transfer behavior.

## 2. Selection of ILs and Preparation of INF

For heat transfer-based applications, ILs are mainly selected based on their thermophysical properties particularly of high thermal conductivity and low viscosity. Another important factor is their miscibility in water due to improving their thermal properties such as thermal conductivity, heat capacity and reducing viscosity.

**Figure 1:**
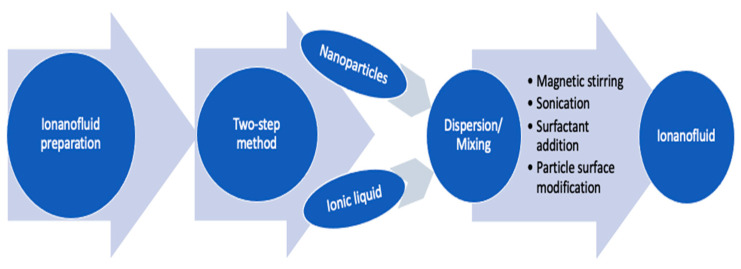
Schematic of concept of ionanofluids preparation.

Preparation of ionanofluids is the first key step, as their properties, performance and suitability in application highly depend on it. The preparation methodology of ionanofluids is similar to those of conventional nanofluids where nanoparticles are either directly synthesized inside the base fluid or mixed in base fluid [16]. While the first route is known as the one-step method, the latter is called the two-step method. For ionanofluids, the one-step method, which is direct synthesis of nanoparticles in base ionic liquid, is rarely used. Whereas, ionanofluids are commonly prepared using the two-step method where dry nanoparticles (purchased or synthesized) are dispersed in base IL and then they are homogenized (better dispersed) mainly using ultra sonication. Schematic of ionanofluids preparation methodology (two-step) is shown in Figure 1, which also highlights different techniques of stable dispersion of nanoparticles in base IL.

Various types of nanoparticles such as Al_2_O_3_, carbon nanotubes, graphene, SiC and graphene oxide are used for the preparation of ionanofluids; whereas, among ionic liquids, imidazolium-based ionic liquids are widely used. While selecting ionic liquid as base fluids for heat transfer-based applications, it is important to choose those with high thermal properties such as thermal conductivity and heat capacity. Although the preparation procedure is quite straight forward, it is very challenging to ensure proper/homogenous dispersion of nanoparticles and long-term stability of prepared ionanofluids. Thus, besides sonication, surfactants are also added to improve stability of prepared ionanofluids. Another way to improve stability is by nanoparticles’ surface treatment or modification. However, the latter option is rarely employed in ionanofluids preparation. It is important to note that special attention must be given while ultrasonicating ionanofluids, as excessive sonication (long time and at high amplitude) can deteriorate the sample in both chemical and physical condition (such as destroying structure and surface of nanoparticles such as CNT). Due to prolong ultrasonication (especially probe type) the sample ionanofluids can be evaporated, and the concentration of nanoparticle can be changed. It is also advisable not to use surfactant, as they can also deteriorate or can become inactive at moderate to high temperature conditions. Nevertheless, it is important to assess the degree of stability of prepared ionanofluids by performing a stability study, which includes determining zeta potential, UV-Vis absorbance, size distribution using dynamic light scattering as well as TEM or SEM analysis. The stability assessments of ionanofluids are the same as commonly used for nanofluids [16,17,18].

## 3. Thermophysical Properties of ILs and INFs Used on Convective Heat Transfer Applications

### 3.1. Ionic Liquids Thermophysical Properties

Thermophysical properties of base ILs are crucial for their own as well as their INFs’ heat transfer performance, particularly for convective heat transfer applications. Thus, important thermophysical properties including viscosity, density, thermal conductivity and heat capacity of commonly considered ILs are presented in Table 1 and Table 2. Reference temperature (mainly room temperature condition) of the property value and corresponding references are also provided. It is noted that the values of these properties can be different in other sources that are not used in these tables. As the focus of this study is not ionic liquid, no analysis of results from individual studies from the literature on these properties of ionic liquid will be provided here. However, a detailed review on ILs thermophysical properties and on ILs as heat transfer fluids can be found elsewhere in the literature (e.g., Chernikova et al. [19]). The data presented in these tables (Table 1 and Table 2) will help to identify suitable ILs for INFs as well as for their applications, particularly in thermal applications.

**Figure 2:**
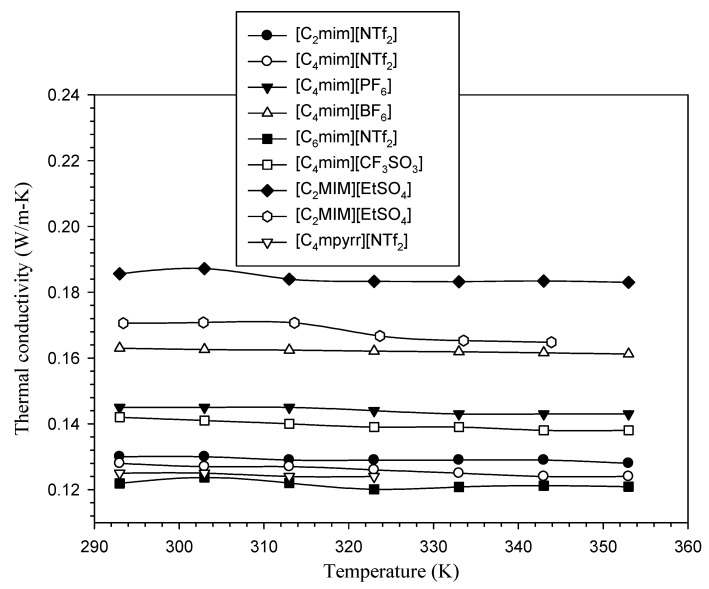
Effect of temperature on the thermal conductivity of ILs [4,48,53,54].

Unlike conventional heat transfer fluids, Figure 2 reveals that temperature does not have noticeable influence on thermal conductivity of ILs. However, as can be seen from Table 2 as well as Figure 2, changing the anion or cation type resulted in a larger variation in thermal conductivity. Varying the alkyl chain length, n, of the [Cnmim][NTf_2_] ionic liquids had no significant effect on the thermal conductivity.

**Figure 3:**
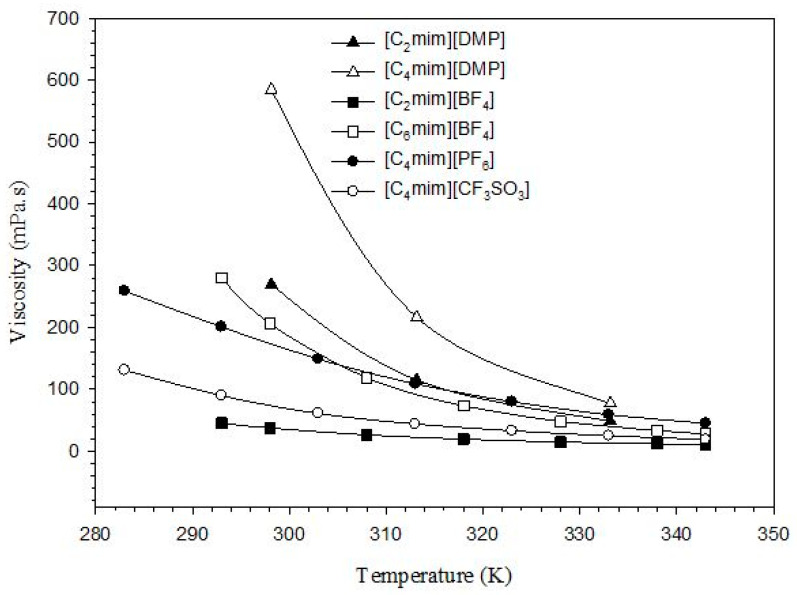
Effect of temperature on the viscosity of ILs [55,56,57].

Ionic liquids commonly exhibit high viscosity and, thus, are not very suitable in convection application. However, similar to common heat transfer fluids, the viscosity of ILs is strongly influenced by temperature, as can be seen from Figure 3 where the viscosity of representative ILs decreases exponentially with the temperature, which is good for their cooling application at high temperature conditions.

Usually, reduction in viscosity and increase in specific heat capacity of ILs are commonly used by mixing with water. For binary mixture of IL and water, the heat capacity of the mixture increases by increasing the mole fraction of water [58]. Heat capacity of IL also increases with temperature [58]. Although there are many immiscible ILs, binary mixture of ILs with water is better as a base fluid for ionanofluids than a heat transfer medium.

### 3.2. Thermophysical Properties of Ionic Liquids-Based Nanocolloids

This section attempts a comprehensive review on thermophysical properties of ionic liquid-based nanocolloids with emphasis on relevant properties for convective heat transfer.

#### 3.2.1. Thermal Conductivity

Thermal conductivity is one of the most important property of fluids when it talks about heat transfer capability of a certain fluid. Table 3 shows experimental data on ionic liquids-based nanocolloids thermal conductivity.

Most of the new fluids contain little concentration of nanoparticles, up to 1 %wt. with several exceptions when the nanoparticles fractions are up to 0.05 (see, for example, Franca et al. [26], Paul [61], Paul et al. [62], Chereches et al. [47,65], Hosseinghorbani et al. [68]). As for base fluids, several ionic liquids were considered and few authors, such as, for example, Xie et al. [37] and Chereches et al. [47,60], made mixtures between water and ILs.

If we consider experimental results on thermal conductivity, all of the authors noticed an increase in thermal conductivity values when nanoparticles are added to the ionic liquids. Nevertheless, the temperature influence was little, as was shown in Table 3. Overall, the enhancement in thermal conductivity is up to 10% at low percentages of nanoparticles. Nevertheless, Ribeiro et al. [59] found an increase of up to 30% when 1%wt. MWCNT are added to several ILs.

For instance, thermal conductivity was found almost constant with temperature variation of several ionic liquids with nanoparticles measured by Franca [26]. The same phenomenon was noticed also by other researchers (see, for example, Ribeiro et al. [59], Patil et al. [60], Ferreira et al. [3] and Ribeiro et al. [4]) concluding that the thermal conductivity of ionic liquids with nanoparticles is following the same trend as that found in the literature for molecular liquids [69] and other ionic liquids [24,53,70,71,72].

Furthermore, a comparison is performed in terms of nanoparticles and/or ionic liquid influence on the thermal conductivity values. From Table 3, we can conclude that the experimental data are scattered and a large variety of combinations were considered. First, the influence of base ionic liquid was checked using two kinds of the most considered nanoparticles: MWCNTs and alumina.

**Figure 4:**
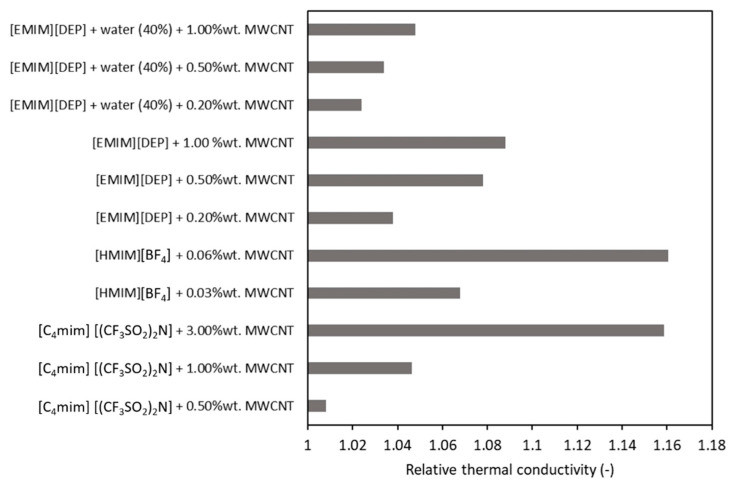
Influence of the base fluid on thermal conductivity of fluids with MWCNTs [26,34,37].

**Figure 5:**
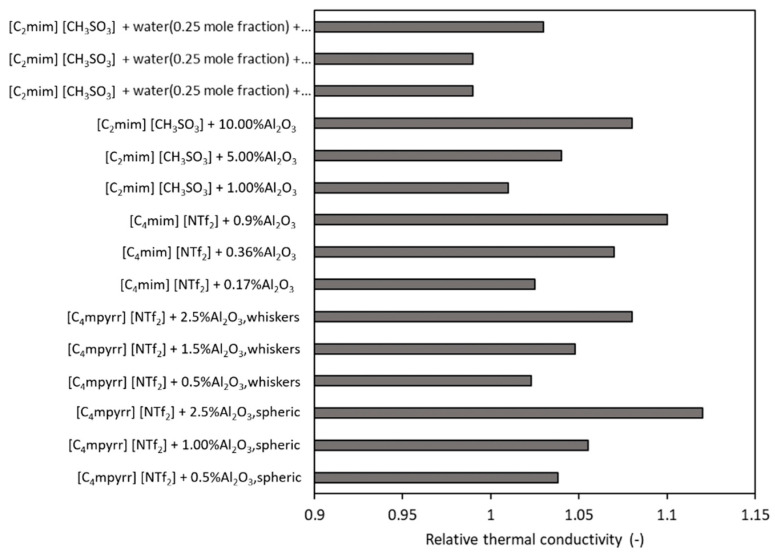
Influence of the base fluid on thermal conductivity of fluids with Al_2_O_3_ nanoparticles.

Figure 4 and Figure 5 synthetize some data from the literature in regard to nanoparticles’ influence on thermal conductivity enhancement if compared with the ionic liquid thermal conductivity. If we look to Figure 4, we can conclude that the enhancement of conductivity is decreasing when a mixture of ionic liquid + water is considered as the base fluid (see the results from Xie et al. [37]). Plus, if we compare data from Franca [26] and Xie et al. [37], we can see that, for the same quantity of nanoparticles, the ionic liquid slightly impacts the experimental values. On the other hand, Wang et al. [34] obtained larger increases with very low quantities of nanoparticles (of 0.03% and 0.06% wt. MWCNT).

Figure 5 contains experimental data using Al_2_O_3_ as a nanoparticle in four base fluids: [C_4_mpyrr][NTf_2_], [C_4_mim][NTf_2_], [C_2_mim][CH_3_SO_3_] and a mixture between [C_2_mim][CH_3_SO_3_] and water (with 0.25 mole fraction) [47,62,63,65]. Results concluded that, for 1%wt. alumina, the thermal conductivity enhancement varies from 0–10%, thus there is a relatively strong influence of the base fluid.

**Figure 6:**
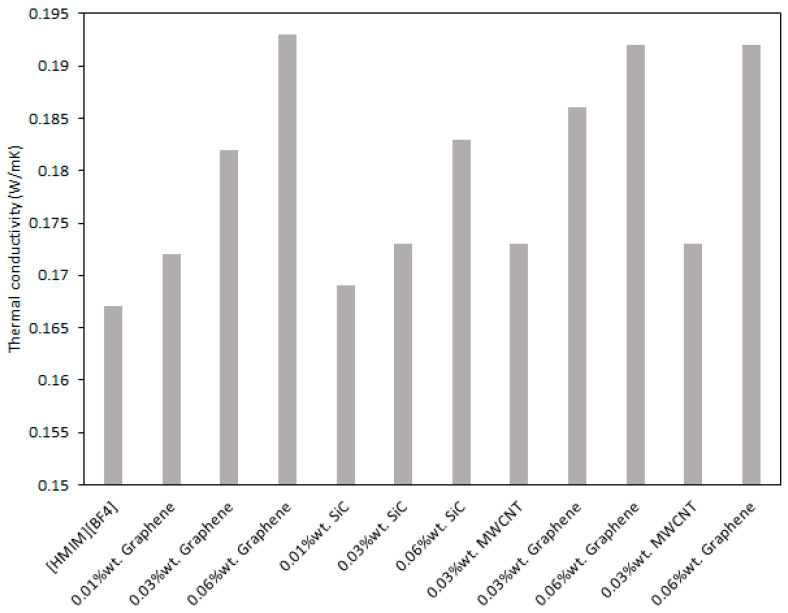
Influence of the nanoparticle type on thermal conductivity of ILs.

Furthermore, in Figure 6, the thermal conductivity values of [HMIM][BF_4_] and of several nanoparticles-enhanced ionic liquids are plotted with the addition of graphene, MWCNT and SiC. Results clearly show that the nanoparticle type influences the experimental conductivity of the fluid. For example, adding 0.03%wt. of graphene, the augmentation is 9%, which is larger than that if SiC or MWCNT are added (i.e., 3.6%).

Concluding, the phenomenon behind the thermal conductivity augmentation is similar with that noticed for regular nanofluids with water or ethylene glycol. Brownian motion seems to be accepted by most of the researchers, while several other mechanisms are discussed in the open literature (for example: thermal boundary resistance, clustering and layering phenomenon), but a number of questions are still unanswered in regard to the main cause for this phenomenon. Another aspect that has to be clarified in the next steps of research is the influence of the base ionic liquid and of the nanoparticle type/shape in order to tailor a better new heat transfer fluid.

#### 3.2.2. Viscosity

While most of nanofluid research has been devoted to thermal conductivity, viscosity has received little attention. Viscosity is a critical parameter when a new fluid for heat transfer applications is developed. This is relevant in the majority of heat transfer applications, where a pumping power is employed to pump the fluids in a certain application.

Most of the experimental studies, as can be seen from Table 4, noticed an increase in viscosity when nanoparticles were added to the ionic liquids, depending on nanoparticles mass concentration (see, for example, Paul et al. [62], Fox et al. [72]). Besides that, several authors (see Patil et al. [60], Ferreira et al. [3], Zhang et al. [68]) found a decrease in viscosity when nanoparticles were added and explained this phenomenon relying on the low density and lubricating properties of nanoparticles, on the interactions between the ions of ILs and the MWCNT, which can hardly be acceptable without a scientific explanation.

**Figure 7:**
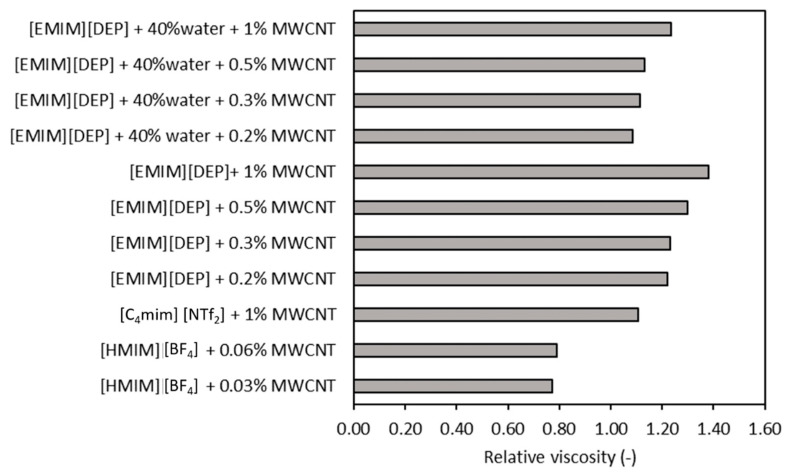
Influence of the base fluid on relative viscosity values of fluids with MWCNTs [34,37,47].

**Figure 8:**
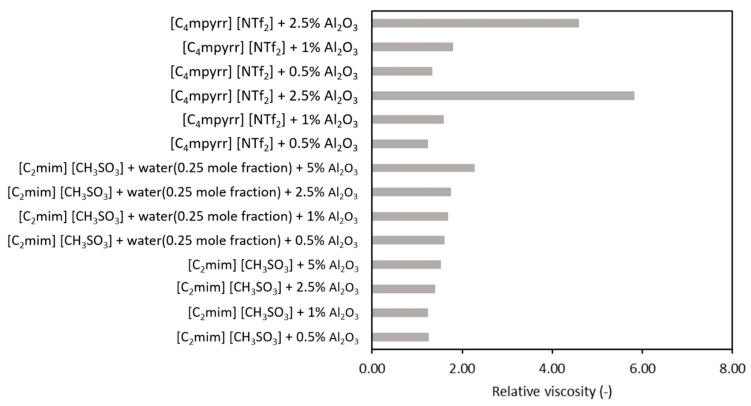
Influence of the base fluid on relative viscosity values of fluids with Al_2_O_3_ [62,65].

Additionally, a comparison is shown in Figure 7 and Figure 8 in terms of nanoparticles and/or ionic liquid influence on viscosity values. From Table 4, it can easily be noticed that the experimental data are scattered.

Figure 7 shows the influence of base ionic liquid using MWCNTs as a base of comparison, and we can conclude that the viscosity is decreasing when a mixture of ionic liquid + water is considered as the base fluid (see the results from Xie et al. [37]). Figure 7 depicts an increase of up to 38% at a small fraction of MWCNTs (i.e., 0.005). Most of the authors found an increase in viscosity when nanoparticles were added to the ionic liquid, and several authors (see, for example, Wang et al. [34]) obtained a decrease. Nevertheless, the decrease in viscosity is a phenomenon rarely noticed and insufficiently described in the literature.

In Figure 8, a comparison for alumina and different base ionic liquid is depicted. A smaller upsurge in viscosity was observed for base fluids from ionic liquids and water mixtures, but the actual influence of the base fluid seems larger at higher nanoparticles’ mass concentrations.

Viscosity increase mechanisms are to be elucidated, and several authors attributed this growth to strong interactions between graphene sheets and IL molecules (see Pamies et al. [26]). Plus, Pamies et al. [38] discussed the increase in concentration based on increases in the internal shear stress, with the subsequent viscosity increase.

Even though in the literature, there are numerous models for viscosity estimation, no theoretical correlation was found acceptable to estimate both nanofluids or other nanoparticle-enhanced fluids’ viscosity behavior. However, a number of papers are proposing the Krieger–Dougherty or Pastorizza–Galllego models (see, for example, the work of Chereches et al. [65] and Pastorizza–Galllego et al. [76]), which seems to describe well the experimental results.

#### 3.2.3. Specific Heat

Specific heat results are also contradictory, as can be clearly seen from Table 5, and it is concluded that the experimental values may greatly depend on the chemical structure of the ionic liquid and of its molecules interaction with nanoparticles.

Based on the previous reports on the simple molecular solvents-based nanofluids, the mechanism of the heat capacity enhancement of ionanofluids is probably driven by the existing interfacial nanolayering occurring on the surface of nanoparticles [45]. 

Zhang et al. [68] found that the decreases noticed for the GNPs-dispersed nanofluids are less than those reached by the SWCNT and GE; the explanation came from the fact that the zero dimensional GNPs has higher thermal energy density than the two-dimensional GE and the one-dimensional SWCNTs.

Some other studies reported the possibility of mesolayers overlapping, as a mechanism of variation of specific heat for nanofluids also extended to the ionic liquids with nanoparticles (see Oster et al. [45]).

In the case of specific heat, since the results are scattered, it is hard to make a good comparison on nanoparticles or ionic liquid influence on the actual variation of the experimental values.

#### 3.2.4. Density

Patil [40] performed some experiments to evaluate the density of several ILs with Ru nanoparticles and noticed a slight decrease in density due to Ru addition, as per Table 6. Overall, the density is the less studied parameter, and all authors concluded that density variation is in line with existing equations, meaning that it increases with nanoparticle addition and decreases with temperature rise.

## 4. Experimental Works on Convective Heat Transfer (for Both ILs and INFs)

Only a handful of experimental works from a single research group on convective heat transfer of ILs and their nanofluids (INFs) are reported in the literature [62,77,78,79,80]. The findings of those works are summarized in Table 7. It can be seen from Table 7 that only Al_2_O_3_ nanoparticle of (three concentrations) was used in three different types of ILs ([N_4111_][NTf_2_], [C_4_mim][NTf_2_]) and [C_4_mpyrr][NTf_2_]), and their convective heat transfer coefficient was determined in forced and natural convection conditions. For laminar flow conditions, they reported a maximum enhancement of heat transfer coefficient of 20% for 1 %wt. loading of spherical shaped Al_2_O_3_ nanoparticle [79]. A natural convection study from the same group [80] showed that whiskers shaped nanoparticles had slightly higher Nu compared to spherical one at the same Ra. However, both nanoparticles actually degraded the natural convection heat transfer. Apart from direct convective heat transfer experimentation, Huminic and Huminic [15] carried out a heat transfer performance analysis using thermophysical properties of [Hmim][SF_4_] and based on nondimensional performance numbers such as the Mouromtseff number as well as calculating a few figures of merit. They concluded that in laminar flow, condition graphene/[Hmim][SF_4_] ionanofluids are beneficial over SiC/[Hmim][SF_4_] in heat transfer applications.

Compared to a relatively large number of numerical works on convection heat transfer of ILs and INFs, such a handful of experimental works was performed due to several reasons among, which are the high price of ILs and nanoparticles as well as ILs and INFs having very high viscosity. Thus, despite showing some enhancement in convection heat transfer of INFs [79,80], based on large pressure drop (leading to high pumping power) and high cost, no conclusions can be made on the suitability of these INFs as advanced heat transfer fluids for convection applications.

## 5. Numerical Works on Convective Heat Transfer of ILs and INFs (for Both ILs and INFs)

One of the first numerical studies performed on ionic liquids and their colloids is from Minea and Murshed [7], who implemented simple geometry into several fully described ionic liquids (i.e., [C_4_mim][NTf_2_] + Al_2_O_3_, [C_4_mim][NTf_2_] + 1%wt. MWCNT; [C_2_mim][EtSO_4_] + MWCNT and [HMIM][BF_4_] + MWCNT/graphene), and the results are depicted in Table 8. One of these authors’ main conclusions is that with increasing flow, the heat transfer coefficient increases considerably, and it appears that the thermal conductivity plays a superior role in laminar convection, while viscosity is of reduced relevance. Plus, heat transfer seems to be greatly influenced by both ionic liquid and nanoparticle type and concentration.

The explanations behind these results are attributed to several phenomenon, such as the increase in viscosity when nanoparticles are added to the ionic liquid; the dominant role of convection over conduction heat transfer when it comes to ionic liquid nanocolloids; the formation of polar molecules (i.e., water molecules) around ionic liquids ions associated with the decrease in bonds between ionic components of the IL when water is added. Furthermore, alumina nanoparticles’ addition marginally drops the ions mobility by substituting water molecules with nanoparticles in the ions vicinity [10,80,81,82].

It may underline here that all the fluids were modelled as single-phase fluids with known thermophysical properties. This is a good approach, especially in the case of experimentally determined properties, as was demonstrated for nanofluids in the only numerical benchmark study, as can be seen from Minea et al. [85]. Of course, other techniques are available, as multiphase model, but no relevant studies were identified in the open literature, where most of the simulations involve calculated properties, based on the nanofluids’ empirical models.

## 6. Theoretical Development and Correlations

In regard to theoretical development of correlations, the literature review revealed little information. Work was performed mostly on simulation and results will be discussed further.

Chereches et al. [10] developed a numerical analysis for laminar and fully developed turbulent flow in a heated circular duct using the single-phase model approach of two ionic liquids ([C_4_mim][NTf_2_] and [C_4_mpyr][NTf_2_]) and three concentrations of nanoparticles (0.5, 1 and 2.5%). A constant heat flux of 12,998.83 W/m^2^ was applied at tube wall. The correlation was developed for the Nusselt number as:

Nu = 4.15 Re^0.09^Pr^0.195^(1 − φ − 200φ^2^). (1)

The correlation, with a ±7% data precision, is valid under the laminar flow regime with 500 < Re < 2000 and total weight concentration ranging from φ = 0 to 2.5%. Based on these results, Chereches et al. [10] found an increase in heat transfer performance and Nu number with the increase in nanoparticle addition, as can also be noticed from Table 8.

Another interesting analysis was performed by El-Maghlany and Minea [11] in a tube subjected to heat flux, with direct application to solar energy. The aforementioned study considered [C_4_mim][NTf_2_] ionic liquid enriched by adding alumina nanoparticles with 0.5, 1 and 2.5% volume concentration. The simulation geometry was similar to the one for the solar collectors, modelling the real application as accurate as possible, and the correlations are (with a deviation of up to 5.5%):

Nu = 0.558 (Re Pr D/L)^0.376^—valid for the ionic liquid, (2)

Nu = 0.6 (Re Pr D/L)^0.372^—valid for φ = 0.5% alumina, (3)

Nu = 0.63 (Re Pr D/L)^0.369^—valid for φ = 1.0% alumina, (4)

Nu = 0.696 (Re Pr D/L)^0.361^—valid for φ = 2.5% alumina. (5)

Another correlation that involves the thermal diffusivity (α) was also proposed by El-Maghlany and Minea [11] as follows:

(6) Nu2.702=0.226Re Pr DL(αfαionano)

Authors explained that the equation reveals the relevant role of thermal diffusivity in evaluating the performance of the heat transfer and concluded that the outcomes show that adding nanoparticles to ionic liquids improves the convection heat transfer, corroborated with low pressure drop consequence.

Another approach comes from studying the ionic liquids and its derivatives in natural convection in a squared enclosure. In this regard, Minea and El-Maghlany [12] performed a study of [C_4_mim][NTf_2_] ionic liquid with small volume concentrations of alumina nanoparticles at Ra = 10^4^–10^6^.

The numerical results are correlated as a function of both Ra and φ, and the results in terms of Nu number are:

- For the hot element at the bottom wall:
  Nu = 81.663 φ + 0.555 (Ra − 4614.793)^0.226^ − 3710.366 φ^2^, (7)
- For the hot element at the left wall:

This is example 1 of an equation:

Nu = 116.173 φ + 0.484 Ra^0.245^ − 5001.894 φ^2^. (8)

Ansarpoura et al. [13] studied [EMIM][EtSO_4_] ionic liquid with small concentrations of alumina nanoparticles in laminar flow and determined a correlation for Nu number using Gauss Newton algorithm using 143 data points and it writes:

(9) Nu=0.772(Re Pr)0.2102 (1+ϕ)−7.721.

The correlation is valid for 500 < Re < 2000, 278.15 < T < 323.15 and for volume concentrations less than 2.5% wt.

Huminic and Huminic [15] performed a very interesting theoretical study on performance evaluation of [Hmim][BF_4_] ionic liquid and several suspensions with nanoparticles (silicon carbide and graphene), using the experimental properties available on the literature. Authors evaluated several figures of merit in laminar and turbulent flows. The conclusion pointed out that ionanofluids can enhance the thermal performance, particularly in laminar flow.

## 7. Conclusions and Future Works

Developing a new heat transfer fluid as well as improving thermal properties of existing ones has become extremely important nowadays due to the necessity of reducing energy consumption in many applications. 

Ionic liquids have major advantages, especially as medium temperature heat transfer fluid, and by adding nanoparticles, the thermal conductivity is augmented resulting in better convective heat transfer coefficients.

Here, an extensive review was performed in terms of properties and thermal convection applications of ionic liquids and their suspensions with nanoparticles. The following conclusions are drawn from this state-of-the-art review:

- Although thermal conductivity of ionic liquids are mostly independent of temperature, viscosity follows the common fluids nature with temperature, as they decrease with temperature;
- Thermal conductivity increases by adding nanoparticles and slowly decreases with temperature;
- Viscosity upsurge depends on nanoparticle addition and type and decreases drastically with increasing temperature;
- Specific heat variation is determined by the type of nanoparticles, while it increases with temperature;
- Density increases with nanoparticle addition and decreases with rising temperature;
- Heat transfer seems to be greatly influenced by both ionic liquid and nanoparticle type and concentration.

Nevertheless, an important drawback of the studies published by now is the lack of insight at a molecular level, such as intermolecular interaction between nanoparticles and the solvent. The phenomenological approach needs to be further developed. Furthermore, the application of artificial intelligence-based predictive methods in ionic liquid studies is at its very beginnings and requires further insights. The first step was noticed in the open literature (see Yusuf et al. [86]), and a number of machine-learning applications in the prediction of several ionic liquids’ properties are carefully reviewed. These predictive methods can also be further extended for the ionic liquids-based nanocolloids; however, a more coordinated approach is recommended.

As a conclusion of this review, it can be inferred that ionic liquids-based nanocolloids can be seen as an efficient method for convective heat transfer enhancement. However, tremendous studies are needed in order to better understand and to elucidate their heat transfer mechanisms together with the interactions between anions, cations and nanoparticles.

## Figures and Tables

**Table 1:** Viscosity and density of ILs used as base fluids for INFs.

Ionic Liquid	Viscosity (mPa·s)	Condition [Reference]	Density (kg/m^3^)	Condition [Reference]
[C_4_mim][NTf_2_]	99.6	298 K [20]	1.436	298 K [1]
[C_6_mim][NTf_2_]	70.5	298 K [21]	1.372	298 K [22]
[C_4_mim][CF_3_SO_3_]	76.0	298 K [23]	1.306	293 K [24]
[C_4_mim][PF_6_]	257.0	298 K [23]	1.372	293 K [24]
[C_6_mim][PF_6_]	485.8	298 K [25]	1.293	298 K [25]
[C_2_mim][EtSO_4_]	125.4	293 K [2]	1.236	298 K [2]
[C_4_mim][(CF_3_SO_2_)_2_N]	51.1	313 K [26]	1.426	313 K [26]
[C_6_mim][BF_4_]	250.0	298 K [27]	1.149	298 K [27]
[C_2_mim][CH_3_SO_3_]	149.1	298 K [28]	1.239	298 K [28]
[N_4111_][NTf_2_]	105.4	298 K [29]	1.392	298 K [30]
[C_4_mpyr][NTf_2_]	68	303 K [31]	1.382	298 K [32]
[(C_6_)_3_PC_14_][NTf_2_]	318	298 K [33]	1.065	298 K [3]
[HMIM][BF4]	250	298 K [34]	1.123	298 K [35]
[C_4_mim][BF4]	85.37	303.15 K [36]	1.198	303.15 K [36]
[EMIM][DEP]	274	298 K [37]	1.148	298 K [37]
[EMIM][DCA]	13.2	300 K [38]	1.1	298 K [39]
[C_4_mim][Cl]	545	333 K [40]	1.087	293 K [40]
[C_4_mim][Br]	215	303 K [40]	1.298	293 K [40]
[C_4_mim][I]	379	303 K [40]	1.489	293 K [40]

**Table 2:** Thermal conductivity and heat capacity of ILs used as base fluids for INFs.

Ionic Liquid	Thermal Conductivity (W/m K)	Condition [Reference]	Heat Capacity (kJ/kg·K)	Condition [Reference]
[C_4_mim][NTf_2_]	0.126	300 K [41]	1.352	298 K [42]
[C_6_mim][NTf_2_]	0.122	293 K [43]	1.426	298 K [22]
[C_4_mim][CF_3_SO_3_]	0.142	293 K [43]	1.484	298 K [44]
[C_4_mim][PF_6_]	0.145	293 K [4]	1.432	308 K [4]
[C_6_mim][PF_6_]	0.142	293 K [24]	1.358	293 K [25]
[C_4_mim][DCA]	0.176	298 K [45]	1.827	296 K [46]
[C_4_mim][BF_4_]	0.163	298 K [43]	1.614	298 K [43]
[C_6_mim][BF_4_]	2.21	298 K [27]	0.166	298 K [27]
[C_2_mim][CH_3_SO_3_]	0.190	298 K [47]	1.629	298 K [47]
[C_2_mim][EtSO_4_]	0.1706	293.4 K [48]	1.57	293 K [49]
[C_4_mim][(CF_3_SO_2_)_2_N]	0.1114	293.4 K [48]	1.373	313 K [26]
[N_4111_][NTf_2_]	0.122	303 K [30]	1.70	303 K [30]
[C_4_mpyrr][NTf_2_]	0.124	303 K [30]	1.58	303 K [30]
[(C_6_)_3_PC_14_)][Phosph]	0.135	298 K [3]	2.12	298 K [3]
[(C_6_)_3_PC_14_][NTf_2_]	0.137	298 K [3]	1.788	333 K [3]
[HMIM][BF4]	0.166	298 K [34]	2.265	298 K [34]
[EMIM][DEP]	0.1749	303 K [37]	1.998	293 K [50]
[C_4_mim][Cl]	0.176	293 K [40]	1.982	298 K [51]
[C_4_mim][Br]	0.16	293 K [40]	1.421	298 K [42]
[C_4_mim][I]	0.131	293 K [40]	1.165	298 K [52]

**Table 3:** Literature results on thermal conductivity of ionic liquid-based nanofluids.

Reference	Ionic Liquid	Nanoparticles	Concentration	Conditions	Observation
Franca et al. [26]	[C_4_mim][(CF_3_SO_2_)_2_N]	MWCNT	0.5–3 %wt.	Temperature variation in the range 293–343 K	1. Thermal conductivity remains almost constant when temperature increases.
	[C_2_mim][EtSO_4_]				2. Thermal conductivity of ionic liquid increases with nanoparticles concentration.
Ribeiro et al. [59]	[C_2_mim][(CF_3_SO_2_)_2_N]	MWCNT	1 %wt.	Temperature variation in the range 293–353 K	1. Thermal conductivity decreases linearly in the studied temperature range.
	[C_4_mim][(CF_3_SO_2_)_2_N]				
	[C_6_mim][(CF_3_SO_2_)_2_N]				2. Thermal conductivity of ionic liquid increases up to 35% when MWCNT are added.
	[C_8_mim][(CF_3_SO_2_)_2_N]				
	[C_4_mim][BF_4_]				
Patil et al. [60]	[C_4_mim][BF_4_]	Ru	0.003 M	Temperature variation in the range 293–333 K	1. Thermal conductivity increase by adding Ru is extremely low—up to 4%.
Ferreira et al. [3]	[(C6)3PC14)][Phosph]	MWCNT	0.05–0.1 %wt.	Temperature variation in the range 283–334 K	1. Thermal conductivity slightly increases, up to 1.5%, with nanoparticle addition.
	[(C6)3PC14)][NTf_2_]				2. Thermal conductivity remains almost constant with temperature.
	[(C6)3PC14)][NTf_2_]				
Paul [61]	[C_4_mpyrr][NTf_2_]	Al_2_O_3_	0.5–2.5%	Temperature variation in the range 303–343 K	Thermal conductivity increases up to 15%, with nanoparticle addition and temperature.
	[C_4_mim][NTf_2_]				
Paul et al. [62]	[N_4_111][NTf_2_]				
Nieto de Castro et al. [24]	[C_4_mim][NTf_2_]	MWCNT	1 %wt.	Room temperature, 293 K	High enrichment (35%) for [C_4_mim][NTf_2_]) + MWCNT and up to 10% rise in thermal conductivity for the other ILs.
	[C_4_mim][CF_3_SO_3_]				
	[C_6_mim][NTf_2_]				
	[C_8_mim][NTf_2_]				
	[C_4_mim][BF_4_]				
Ribeiro et al. [4]	[C_6_mim][BF_4_]	MWCNT	1 %wt.	Temperature variation in the range 293–353 K	1. A moderate increase in the thermal conductivity was noticed when temperature rises.
	[C_4_mim][CF_3_SO_3_]				
	[C_4_mpyrr][NTf_2_]				2. Thermal conductivity is enhanced up to 10% when MWCNT are added.
	[C_4_mim][PF_6_]				
	[C_6_mim][PF_6_]				
Wang et al. [34]	[HMIM][BF_4_]	Graphene MWCNT	0.03 and 0.06 %wt.	Temperature variation in the range 298–338 K	1. Enhancement of up to 20% at nanoparticle addition.
					2. Temperature has little to no influence.
Jorjani et al. [63]	[BMIM][BF_4_]	Nano-diamond	0.36–1.04 %vol.	Ambient temperature	1. Enhancement of up to 9.3% at nanoparticle addition.
Liu et al. [35]	[HMIM][BF_4_]	Graphene	0.03, 0.06 %wt.	Ambient temperature	1. Thermal conductivity increases up to 13.1% at 0.06 %wt.
					2. Thermal conductivity increases with temperature.
Xie et al. [37]	[EMIM]	MWCNT	0.2, 0.5, 1 %wt.	Ambient temperature	Thermal conductivity increases up to 9.7%.
	[DEP] + DI water				
Paul et al. [64]	[C_4_mim][NTf_2_]	Al_2_O_3_	0.18, 0.36, 0.9 %vol.	Ambient temperature	Thermal conductivity increases by 11% for 0.9 %vol.
Chereches et al. [47,65]	[C_2_mim][CH_3_SO_3_]	Al_2_O_3_	0.5–5 %wt.	Temperature variation in the range 293–353 K	1. Thermal conductivity increases by 12.9% when alumina is added.
					2. Thermal conductivity variation with temperature is not significant.
Chereches et al. [47,65]	[C_2_mim][CH_3_SO_3_] + water	Al_2_O_3_	0.5–5 %wt.	Temperature variation in the range 293–353 K	1. Thermal conductivity increases up to 10% when alumina is added.
					2. Thermal conductivity variation with temperature is not significant.
Chen et al. [66]	[HMIM][BF_4_]	SiC	0.01, 0.03 and 0.06 %wt.	Temperature variation in the range 298–358 K	1. Thermal conductivity increases up to about 10% when SiC is added.
					2. Thermal conductivity increases with temperature.
Jorjani et al. [63]	[BMIM][BF_4_]	Nano-diamond	0.36, 0.69 and 1.04 %vol.	Ambient temperature	Thermal conductivity enhancement percentages of 4.2, 5.3 and 9.3 if compared to the base fluid and in respect to increasing the volume fraction of the nanodiamond.
Hosseinghorbani et al. [67]	[Bmim][NTf_2_]	graphene oxide (GO)	0.5, 1, 2 %wt.	Temperature variation in the range 288–328 K	Thermal conductivity increases with temperature. The enhancement is up to 6.5% at 2% mass concentration of GO nanoparticles.
Zhang et al. [68]	[BMIM][BF_4_]	GNP, SWCNT, graphene	0.005, 0.01 %wt.	Temperature variation in the range 293–428 K	At ambient temperature, thermal conductivity increases with nanoparticle addition, while graphene influence is higher.
					When temperature rises to 428 K, thermal conductivity enhancement is up to 16.3%, depending on nanoparticle type and concentration.
Xie et al. [37]	[EMIM][DEP]	MWCNT	1 %wt.	Temperature variation in the range 298–353 K	Thermal conductivity increases within the range of 1.3–9.7% compared to ionic liquids.
	[EMIM][DEP] + H_2_O				Temperature influence is a linear one.

**Table 4:** Literature results on viscosity of ionic liquid-based nanofluids.

Reference	Ionic Liquid	Nanoparticles	Concentration	Conditions	Observation
Patil et al. [60]	[C_4_mim][BF_4_]	Ru	0.003 M	Temperature variation in the range 303–373 K	1. The viscosities of ILs and INFs reduce substantially with temperature increase.
	[C_4_mim][Cl]				
	[C_4_mim][Br]				2. The viscosity of ILs decreases significantly with the addition of Ru particles.
	[C_4_mim][I]				
Ferreira et al. [3]	[(C_6_)_3_PC1_4_)][Phosph]	MWCNT	0.05–0.1 %wt.	Temperature variation in the range 283–334 K	1. The viscosities of ILs and INFs reduces with temperature increase.
	[(C_6_)_3_PC1_4_)][NTf_2_]				2. The viscosity of ILs decreases significantly with the addition nanoparticles.
	[(C_6_)_3_PC1_4_)][NTf_2_]				
Wang et al. [34]	[HMIM][BF_4_]	Graphene MWCNT	0.03 and 0.06 %wt.	Temperature variation in the range 298–348 K	1. The viscosities of ILs and INFs remain almost constant with temperature increase.
					2. The viscosity of ILs decreases with the addition nanoparticles.
Paul et al. [62]	[C_4_mpyrr][NTf_2_]	Al_2_O_3_	0.5–2.5%	Temperature variation in the range 293–353 K	1. The viscosities of ILs and INFs decreases with temperature increase.
					2. The viscosity of ILs increases significantly with the addition of nanoparticles, up to 600%.
					3. The viscosity variation also depends on the nanoparticle shape (whiskers NP gives lower viscosity results if compared with spherical nanoparticles).
Fox et al. [72]	[C_4_mmim][NTf_2_]	SiO_2_	0.5 %wt.	Ambient temperature 298 K	1. Viscosity increases when nanoparticles are added to the ionic liquid. The increase varies from 3% (for SiO_2_) up to 52% (for CB)
		Au			
		ZnO			
		CuO			
		Fe_2_O_3_			2. The viscosity variation also depends on the nanoparticle type.
		SGNF (stacked graphene nanofiber)			
		MWCNT			
		CB (carbon black)			
Jorjani et al. [63]	[BMIM][BF_4_]	Nanodiamond	0.36–1.04 %vol.	Ambient temperature	1. Increase between 32 and 126% when nanoparticles are added.
Paul et al. [64]	[C_4_mim][NTf_2_]	Al_2_O_3_	0.18, 0.36, 0.9 %vol.		Shear viscosity of ionanofluid decreases with the rise in shear rate where shear thinning occurred.
Chereches et al. [65]	[C_2_mim][CH_3_SO_3_]	Al_2_O_3_	0.5–5 %wt.	Temperature variation in the range 293–353 K	1. Viscosity increases between 39 to 78% when alumina is added.
	[C_2_mim][CH_3_SO_3_] + water				2. Viscosity decreases with temperature.
Alizadeh and Moraveji [73]	[BMIM][PF_6_]	GNP	1–3 %wt.	Temperature range: between 293.15 and 333.15 K.	1. Viscosity reduces as temperature rises.
					2. At 293.15 K, viscosity of ionanofluids containing 1, 2 and 3% wt. GNPs are around 20, 27 and 43% lower than that of pure ionic liquid.
					3. The relative viscosity increases with enhancement of temperature.
Chen et al. [66]	[HMIM][BF_4_]	SiC	0.01, 0.03 and 0.06 %wt.	Temperature variation in the range 298–358 K	1. The viscosity decrease nonlinearly with the increasing temperature, where the viscosity of 0.03 %wt. SiC fluids decreases from 275 to 67 cp as the temperature increases up to 358 K.
					2. Nanoparticles loading induces the viscosity increase in fluids, where the viscosity value at 298 K increases from 250 to 289 cp.
Hermida-Merino et al. [74]	[C2C1py][C_4_F_9_SO_3_]	GNP	1, 5 and 10 %wt.	Temperature variation in the range 293–353 K	Viscosity decreases with temperature and increases with nano additive concentration.
Pamies et al. [38]	[EMIM][TFSI]	graphene	0.5, 1 %wt.	Temperature variation in the range 298 to 393 K	[EMIM][DCA] shows much lower viscosity values than [EMIM][TFSI], and an increase in graphene content increases the viscosity values, but this increase is higher in the case of [EMIM][TFSI]. The increase is between 48.5–269% depending on the ionic liquid type and nanoparticle loading. The decrease in viscosity appears with increasing temperature.
	[EMIM][DCA]				
Jorjani et al. [67]	[BMIM][BF_4_]	Nanodiamond	0.36, 0.69 and 1.04 %vol.	Ambient temperature	The viscosity increase percentages were 32, 67 and 126, if compared to the base fluid and in respect to increasing the volume fraction of the nanodiamond.
Soman et al. [75]	[BMIm][Br]	Al_2_O_3_	0.1 to 0.6 %wt.	Temperature variation in the range 293.15 to 373.15 K	Viscosity of aqueous 1-butyl-3-methylimidazoliumbromide suspensions increases with concentration and decreases with temperature.
Hosseinghorbani et al. [67]	[Bmim][NTf_2_]	graphene oxide (GO)	0.5, 1, 2 %wt.	Temperature variation in the range 298–348 K	The shear stress data were obtained for shear rates between 3.96 and 79.2 s^−1^ at 298 K.
					As the concentration of nanoparticles increases, the viscosity increases. When concentration amplifies from 1 to 2%, the viscosity changes from 68.8 to 180 cP at room temperature.
					Increasing the temperature decreases viscosity.
Zhang et al. [68]	[BMIM][BF_4_]	GNP, SWCNT, graphene	0.005, 0.01 %wt.	Temperature variation in the range 293–428 K	Viscosity decreases drastically with temperature increase.
					Viscosity also decreases when nanoparticles are added to the base fluid, maximum decrease being attained for lower concentrations.
Xie et al. [37]	[EMIM][DEP]	MWCNT	0.2, 0.5, 1 %wt.	Temperature variation in the range 298–323 K	The viscosity is reduced when the amount of water in the base fluid is increased.
	[EMIM][DEP] + H_2_O				Viscosity increases with increasing volume fraction of the MWCNTs and decreases with temperature.

**Table 5:** Literature results on specific heat of ionic liquid-based nanofluids.

Reference	Ionic Liquid	Nanoparticles	Concentration	Conditions	Observation
Paul [61]	[C_4_mpyrr][NTf_2_]	Al_2_O_3_	0.5–2.5%	Temperature variation in the range 293–353 K	Specific heat increases up to 65%, with nanoparticle addition while temperature influence is small.
	[C_4_mim][NTf_2_]				
Paul et al. [62]	[N_4_111][NTf_2_]				
Wang et al. [34]	[HMIM][BF_4_]	Graphene	0.03 and 0.06 %wt.	Temperature variation in the range 293–353 K	1. Decrease of up to 3% at nanoparticle addition.
		MWCNT			2. Temperature has little to no influence.
Paul et al. [64]	[C_4_mim][NTf_2_]	Al_2_O_3_	0.18, 0.36, 0.9 %vol.	Ambient temperature	Heat capacity increases by 49% for 0.9 %vol.
Chereches et al. [48,65]	[C_2_mim][CH_3_SO_3_]	Al_2_O_3_	0.5–5 %wt.	Temperature variation in the range 293–353 K	Isobaric specific heat capacity is found to decrease with mass fraction and to increase with temperature.
	[C_2_mim][CH_3_SO_3_] + water				
Chen et al. [66]	[HMIM][BF_4_]	SiC	0.01, 0.03 and 0.06 %wt.	Temperature variation in the range 298–358 K	1. Specific heat increases up to 4% at nanoparticle addition, at ambient temperature.
					2. Specific heat increases up to 9% at temperature growth.
Hermida-Merino et al. [74]	[C_2_C1py][C_4_F_9_SO_3_]	GNP	1, 5 and 10 %wt.	Temperature variation in the range 293–353 K	Specific heat increases with both nanoparticle addition and temperature.
Oster et al. [5]	[C_4_C1Im][Dca]	Carbon nanotubes, Boron nitride, Graphite	0.5–3 %wt.	Temperature range was set from 298.15 to 363.15 K.	Heat capacity enhancement is determined by the type of nanoparticles, instead of type of ionic liquid.
	[C_4_C1Im][NTf_2_]				
	[C_2_C1Im][C_2_SO_4]_				Heat capacity increases with temperature.
	[C_4_C1Pyrr][NTf_2_]				
	[C_6_C1Im][PF_6_]				
Hosseinghorbani et al. [67]	[Bmim][NTf_2_]	graphene oxide (GO)	0.5, 1, 2 %wt.	Temperature variation in the range 288–348 K	Specific heat capacity increases when temperature rise. Specific heat capacity enhances up to 42% at 2% mass fraction of GO nanoparticles.
Zhang et al. [68]	[BMIM][BF_4_]	GNP, SWCNT, graphene	0.005, 0.01 %wt.	Temperature variation in the range 293–428 K	Specific heat variation is determined by the type of nanoparticles.
					Specific heat increases with temperature and decreased when nanoparticles are added.

**Table 6:** Literature results on density of ionic liquid-based nanofluids.

Reference	Ionic Liquid	Nanoparticles	Concentration	Conditions	Observation
Patil et al. [60]	[C_4_mim][Cl]	Ru	0.003 M	Temperature variation in the range 293–333 K	1. Density increase by adding Ru is up to 50%.
	[C_4_mim][Br]				
	[C_4_mim][I]				2. Density decreased when temperature rises.
	[C_4_mim][BF_4_]				
Chereches et al. [47]	[C_2_mim][CH_3_SO_3_]	Al_2_O_3_	0.5–5 %wt.	Temperature variation in the range 293–353K	Density is found to be in line with existing equations. Density increases with nanoparticle addition and decreased with temperature.
	[C_2_mim][CH_3_SO_3_] + water				
Chen et al. [66]	[HMIM][BF_4_]	SiC	0.01, 0.03 and 0.06 %wt.	Temperature variation in the range 298–358 K	1. Density increase by adding SiC from 1.14 to 1.21 g/cm^3^.
					2. Density decreases when temperature rises.
Oster et al. [5]	[C_4_C1Im][Dca]	Carbon nanotubes, boron nitride, graphite	0.5–3 %wt.	Temperature range set from 298.15 to 363.15 K.	Density is found to be in line with existing equations. Density increases with nanoparticle addition and decreases with temperature.
	[C_4_C1Im][NTf_2_]				
	[C_2_C1Im][C_2_SO_4_]				
	[C_4_C1Pyrr][NTf_2_]				
	[C_6_C1Im][PF_6_]				
Jorjani et al. [63]	[BMIM][BF_4_]	Nanodiamond	0.36, 0.69 and 1.04 %vol.	Ambient temperature	Density is found to be in line with existing equations. Density increases with nanoparticle addition and decreases with temperature.
Hosseinghorbani et al. [67]	[Bmim][NTf_2_]	graphene oxide (GO)	0.5, 1, 2 %wt.	Temperature variation in the range 298–338 K	Density increases with nanoparticle addition and decreases with temperature.
Xie et al. [37]	[EMIM][DEP]	MWCNT	0.2, 0.5, 1 %wt.	Temperature variation in the range 298–323 K	Density increases with nanoparticle addition and decreases with temperature.
	[EMIM][DEP] + H_2_O				

**Table 7:** Summary of experimental studies on convective heat transfer of ILs and their nanofluids from the literature.

Reference	IL	Nanoparticles	Concentration	Geometry	Type of Convection/Flow Regime	Findings
Paul et al. [77]	[C_4_mmim][NTf_2_]	-	-	Rectangular enclosure	Natural convectionLaminar	Nusselt number of IL is found to be higher (42%) than that of DI water.
					(Ra = 1.13 × 10^7^ − 7.7 × 10^7^)	
Paul et al. [78]	[N_4111_][NTf_2_]	-	-	Circular tube	Forced convection	Nu of this IL is found to well correlate with well-known Shah’s and Gnielinski’s equations.
					Laminar and turbulent (Re: 512–1955 and Re: 3220–5333)	
Paul et al. [79]	[C_4_mim][NTf_2_]	Al_2_O_3_	1 %wt.	Circular tube	Forced convection/laminar flow	Maximum 20% enhancement in convective heat transfer performance.
	[C_4_mpyrr][NTf_2_]	(spherical shape)				
Paul et al. [80]	[N_4111_][NTf_2_]	Al_2_O_3_	0.5 %wt.	Circular tube	Forced convection/laminar flow	15% enhancement in heat transfer performance.
		(spherical shape)				
Paul et al. [62]	[C_4_mpyrr][NTf_2_]	Al_2_O_3_	0.5, 1, 2.5 %wt.	Rectangular enclosure	Natural convection/laminar	Although IL with whiskers -shaped nanoparticles shows slightly higher Nu compared to spherical one at the same Ra, both nanoparticles are actually found to degrade the natural convection heat transfer.
		(spherical and whiskers shapes)				

**Table 8:** Results on numerical implementation of ionic liquids-based nanofluids.

Reference	Ionic Liquid	Nano Particles	Geometry	CFD Code	Flow Type	HTC Enhancement
Minea and Murshed [7]	[C_4_mim][NTf_2_]	Al_2_O_3_	Tube	Ansys Work bench	Steady, laminar forced flow	At Re = 2000, an enhancement of up to 55.6%, depending on NP concentration
	[C_4_mim][NTf_2_]	MWCNT	Tube	Ansys Work bench	Steady, laminar forced flow	At Re = 2000, an enhancement of 11.1% for 1% wt. MWCNT
	[C_2_mim][EtSO_4_]	MWCNT	Tube	Ansys Work bench	Steady, laminar forced flow	At Re = 2000, an enhancement of 8.5% for 1% wt. MWCNT
	[HMIM][BF_4_]	MWCNT Graphene	Tube	Ansys Work bench	Steady, laminar forced flow	At Re = 2000, an enhancement of up to 12.1%, depending on NP concentration or type. Higher values were attained for graphene.
Chereches et al. [10]	[C_4_mim][NTf_2_]	Al_2_O_3_	Tube	Ansys Work bench	Steady, laminar/turbulent forced flow	Enhancement of heat transfer coefficient up to 619.7% is noticed when Re increases and alumina nanoparticles are added to the base ionic liquid, and this enrichment is as high as the Al_2_O_3_ concentration increases.
	[C_4_mpyrr][NTf_2_]					
Chereches et al. [81,82]	[C_2_mim][CH_3_SO_3_]	Al_2_O_3_	Two zone tube	Ansys Work bench	Steady, laminar forced flow	The convective heat transfer coefficient is decreasing up to 70% when water is added to the ionic liquid.
						The increase in Re from 500 to 2000 determines an upsurge of the convection heat transfer coefficient up to about 13%.
	[C_2_mim][CH_3_SO_3_] + water					NEILs heat transfer coefficient goes to an augmentation of up to 50% by adding alumina nanoparticles in the 0.25W + 0.75IL mixture.
El-Maghlany and Minea [11]	[C_4_mim][NTf_2_]	Al_2_O_3_	Tube	In-house code using the finite volume technique	Re = 100–2000	The nanoparticles addition improves the heat transfer with low pressure drop penalty.
					Laminar flow with longitudinal and radial flow (no swirl flow) simulating solar application	
Minea and El-Maghlany [12]	[C_4_mim][NTf_2_]	Al_2_O_3_	Square enclosure	In-house code using the finite volume technique	Natural convection	Nu number varies slightly with the temperature increase and volume concentration of alumina nanoparticles.
Dayf et al. [83]	[C_4_mim][NTf_2_]	Al_2_O_3_	Cubic cavity	In-house code using the finite volume method	Natural convection	The addition of nanoparticles allows a noteworthy increase in heat transfer compared to the base fluid.
Liu et al. [84]	[HMIM][BF_4_]	Graphene	Cylindrical receiver	MAT LAB		The receiver efficiency increases with increasing solar concentration and receiver height, but conversely with the graphene concentration under concentrated incident solar intensity.
Ansarpour et al. [13]	[EMIM][EtSO_4_]	Al_2_O_3_	Tube	Fluent 16.2	Laminar flow	The enhancement in heat transfer coefficient was up to 44.9% by adding nanoparticles.

## Abbreviations

[C_4_mim][NTf_2_]	1-butyl-3-methylimidazolium bis(trifluoromethylsulfonyl)imide
[(C_6_)_3_PC_14_)][Phosph]	Trihexyltetradecylphosphoniumphosphinate
[(C_6_)_3_PC_14_][NTf_2_]	Trihexyltetradecylphosphoniumbis(trifluoromethylsulfonyl)imide
[C_2_mim][CH_3_SO_3_]	1-Ethyl-3-methylimidazolium methanesulfonate
[C_2_mim][EtSO_4_]	1-ethyl-3-methyl imidazoliumethylsulfate
[C_4_mim][(CF_3_SO_2_)_2_N]	1-n-butyl-3-methylimidazolium bis(trifluoromethanesulfonylimide)
[C_4_mim][BF_4_]	1-Butyl-3-methylimidazolium tetrafluoroborate
[C_4_mim][Br]	1-butyl-3-methylimidazolium bromide
[C_4_mim][CF_3_SO_3_]	1-n-butyl-3-methylimidazoliumtrifluoromethanesulfonate
[C_4_mim][Cl]	1-n-butyl-3-methylimidazolium chloride
[C_4_mim][DCA]	1-n-butyl-3-methylimidazolium dicyanamide
[C_4_mim][I]	1-butyl-3-methylimidazolium iodide
[C_4_mim][NTf_2_]	1-butyl-3-methylimidazolium bis(trifluoromethylsulfonyl)imide
[C_4_mim][PF_6_]	1-butyl-3-methylimidazolium hexafluorophosphate
[C_4_mpyr][NTf_2_]	N-butyl-N-methylpyrrolidiniumbis(trifluoromethanesulfonyl)imide
[C_6_mim][BF_4_]	1-hexyl-3-methylimidazolium tetrafluoroborate
[C_6_mim][NTf_2_]	1-hexyl-3-methylimidazolium bis(trifluoromethylsulfonyl)imide
[C_6_mim][PF_6_]	1-hexyl-3-methylimidazolium hexafluorophosphate
[EMIM][DCA]	1-Ethyl-3-Methylimidazolium dicyanamide
[EMIM][DEP]	1-Ethyl-3-Methylimidazolium Diethyl Phosphate
[HMIM][BF_4_]	1-Methylimidazolium tetrafluoroborate
[N_4111_][NTf_2_]	butyltrimethylammoniumbis(trifluoromethylsulfonyl)imide
